# MicroRNA-497 inhibits cellular proliferation, migration and invasion of papillary thyroid cancer by directly targeting AKT3

**DOI:** 10.3892/mmr.2022.12843

**Published:** 2022-09-05

**Authors:** Juhua Zhuang, Ying Ye, Guoyu Wang, Jing Ni, Saifei He, Cuihua Hu, Wei Xia, Zhongwei Lv

Mol Med Rep 16: 5815–5822, 2017; DOI: 10.3892/mmr.2017.7345

Following the publication of this paper, it was drawn to the Editors’ attention by a concerned reader that certain of the data shown for the cell migration and invasion assays in Figs. 2C and 5C were strikingly similar to data appearing in different form in other articles by different authors. Owing to the fact that the contentious data in the above article had already been published elsewhere, or were already under consideration for publication, prior to its submission to *Molecular Medicine Reports*, the Editor has decided that this paper should be retracted from the Journal. The authors were asked for an explanation to account for these concerns, but the Editorial Office did not receive a satisfactory reply. The Editor apologizes to the readership for any inconvenience caused.

